# Complementary and alternative medicine use by pregnant women in Japan: a pilot survey

**DOI:** 10.1186/s12906-023-04126-1

**Published:** 2023-08-24

**Authors:** Ayana Watanabe, Satomi Inoue, Aiko Okatsu, Hiromi Eto, Michiko Oguro, Yaeko Kataoka

**Affiliations:** 1 St. Luke’s International Hospital, 9-1 Akashi-Cho, Tokyo, 104-8560 Japan; 2 St. Luke’s International University, 10-1, Akashi-Cho, Chuo-Ku, Tokyo, 104-0044 Japan; 3 Japanese Red Cross Toyota College of Nursing, 12-33 Nanamagari, Hakusan-Cho, Toyota, Aichi, 471-8565 Japan; 4 Nagasaki University, Institute of Biomedical Sciences, 1-7-1 Sakamoto, Nagasaki, 852-8520 Japan; 5 Tokyo Healthcare University, 1-1042-2 Kaijincho Nishi, Funabashi City, Chiba 273-8710 Japan

**Keywords:** Complementary and alternative medicine, Pregnancy, Therapy, Self-care, Survey

## Abstract

**Background:**

Complementary and alternative medicine (CAM) are popular among women, and are used during their pregnancy in Japan. This study aimed to survey the prevalence of CAM use by healthy pregnant women as a health-care prescribed therapy or self-care and to investigate the factors associated with CAM use in Japan.

**Methods:**

In this cross-sectional study, pregnant women after 34 weeks of gestation were asked to respond to a questionnaire at the clinic or online. The questionnaire comprised questions on the participants’ characteristics and their use of CAM for therapy and self-care. Descriptive statistics were calculated in the analyses, and bivariate and multivariate logistic analyses were performed to evaluate the associations between factors and CAM use.

**Results:**

A total of 394 women responded from three hospitals, two clinics, and two midwifery birth centers. CAM was received as treatment by practitioners during pregnancy by 75 women (19.0%). The following therapies were used: traditional Chinese medicine (7.9%), chiropractic (6.9%), moxibustion (6.4%), and acupuncture (5.3%). One or more types of therapy were used as self-care by 348 women (88.3%). Highly used CAM for self-care were: folic acid supplementation (75.4%), other supplements (51.5%), herbs (20.8%), and yoga (19.0%). Multiple logistic regression analyses revealed that the factors associated with CAM use as a therapy were midwifery birth centers for planned childbirth settings (adjusted odds ratio [AOR] 3.64, 95% confidence interval [CI] [1.69–7.83]) and pregnancy complications diagnosed (AOR 2.46, 95%CI [1.38–4.39]). The factors associated with CAM use for self-care were age 30–39 years (AOR 4.48, 95%CI [2.14–9.73]) and over 40 years (AOR 3.92, 95%CI [1.10–13.91]), junior college education or above (AOR 2.30, 95%CI [1.18–4.51]), and primiparas (AOR 3.82, 95%CI [1.86–7.86]). The most common source of information was the “Internet” (43.8%).

**Conclusions:**

Approximately 20% of Japanese pregnant women received CAM as therapy by practitioners, and the related factors were: tended to have baby at midwifery birth center and pregnancy complications. Almost 90% of respondents used CAM as self-care and the related factors were: older, had a higher educational level and tended to be primiparas. They used the Internet as their main source of information about CAM. Health care providers need to provide evidenced-based information on CAM and to help decision making to ensure safe and effective CAM utilization by pregnant women.

## Background

The development of contemporary medicine has mainly been centered on conventional medicine, especially the technical and evidence-based advancements in medical care [1]. Concurrently, treatment and therapies other than conventional medicine, such as traditional medicine, with its long history, are the sum of the knowledge, skills, and practices based on the theories, beliefs, and experiences indigenous to different cultures [2]. These traditional healthcare practices and others have been categorized as complementary and alternative medicines (CAM). The National Center for Complementary and Integrative Health (NCCIH) in the United States differentiates between the terms “complementary” medicine and “alternative” medicine [3].

Complementary medicine is not a complete system of care and thus can be easily used in conjunction with conventional medicine. Complementary therapies are categorized as natural products, mind and body practices, and other complementary health approaches [3]. Common examples include supplements, mindfulness, massage, *shiatsu*, and the use of essential oils. The NCCIH classified them by their primary therapeutic input: nutritional, psychological, physical, or in combination [3]. Generally, these therapies do not require a physician order or prescription. Recently, conventional and complementary approaches provided together in a coordinated way is termed integrative medicine [3].

The term “alternative medicine” refers to a complete medical care system that is used in place of conventional medicine (also called mainstream or allopathic medicine) [3]. Common examples are homeopathy, naturopathy, traditional Chinese medicine, which includes acupuncture, moxibustion, and herbs, *ayurvedic* medicine from India, and traditional Japanese herbal medicine (*kampo*). In Japan, integrative medicine is a combination of conventional medicine, traditional medicine and complementary and/or alternative medicine to improve patient’s quality of life (QOL) depending on their needs. It is based on the premises of contemporary conventional medicine and led by physicians, along with the help of a multidisciplinary team, as necessary [4]. For the purposes of this paper, we refer to CAM as including any or all of those CAM approaches.

CAM has been used as a safe alternative to pharmaceuticals during the perinatal period, or for treating various symptoms and problems arising during pregnancy. Consequently, CAM allows women greater choice and control over their pregnancy and childbearing experiences [5]. Moreover, many of these therapies were also used as self-care to maintain or promote the well-being of pregnant women and their fetuses. Treatments such as exercise for relieving lower back pain and pelvic pain [6], reflexology and water immersion for leg edema [7], acupuncture and moxibustion for the breech position [8, 9], and ginger for nausea and vomiting [10] have revealed some effectiveness. Furthermore, therapies such as acupuncture and massage have been effective for reducing maternal anxiety and depression [11]. However, strong evidence to support the effectiveness and safety of CAMs is still lacking [12].

The use of CAM during pregnancy is common worldwide. Surveys have found that, in the United States, 69% of pregnant women were using CAM [13], in the United Kingdom 57% [14], and in Germany 51% [15]. In Palestine the most popular treatments were supplements and herbs [16]. In Germany, acupuncture ranked highest at 29.8%, followed by homeopathy (18.5%), phytotherapy (15.1%), and massage (12.2%) [15]. In Nigeria, the most commonly used practices were traditional birth attendants (TBA), followed by herbal mixtures, and the herbal tea *“mvuruinu*” [17]. In a Scottish survey, the reported order of use was herbs, massage, and yoga [18]. In Japan, surveys have been conducted on the use of each therapy, such as the intake of folic acid [19] and supplements [20], but no comprehensive survey on the prevalence of CAM used by pregnant women has been reported.

Several literature reviews have reported an association between the demographic and obstetric characteristics of pregnant women and CAM use [5, 21, 22]. Most studies have found that educational background and parity are associated with CAM use [5]. Moreover, older age, higher education and income, and more physical symptoms were revealed as factors related to the use of complementary products or therapies during pregnancy [21]. The independent predictors of CAM use were CAM use prior to pregnancy, higher education, chronic disease, and ethnic background/nationality [22]. Currently the characteristics of Japanese pregnant women who use CAM are unknown. They need to be identified in order to provide focused advice concerning CAM and appropriate support for decision making about their use of CAM for self-care. However, little is known about the use of CAM and associate factor among Japanese women during pregnancy.

Thus, this pilot study aimed to determine the prevalence of CAM use by pregnant women for therapy and self-care in Japan, reasons for each CAM use, main sources of CAM use, characteristics of women associated with CAM use, and CAM therapies of interests using an original self-report questionnaire.

## Methods

### Study design and participants

This cross-sectional study used anonymous self-report questionnaires. Participants who met the inclusion criteria of this study were healthy pregnant women, past 34 weeks of gestation, and who were able to read and write Japanese. Pregnant women who had mental health problems or serious pregnancy complications (conditions that required hospitalization or bed rest) were excluded. The cooperating institutions included three hospital, two clinics, and two midwifery birth centers in Japan.

A sample size of 383 was recommended for a margin of error of 5%, confidence interval (CI) of 95% using the approximate number of births (pregnant women) per year in Tokyo, which was 100,000 [23] according to the sample size calculation [24]. Tokyo was selected as most of the participating facilities were located in Tokyo. We assumed a 50% response rate [25], so the sample size was increased to 580 participants.

### Survey questionnaire

An anonymous questionnaire in Japanese was developed by the researchers. The questionnaire included three sections. The first was a section on the demographic and obstetric characteristics of participants. Demographic characteristics included age, educational level, occupation, marital status, and current residence. Obstetric characteristics included gravidity, gestational weeks, planned location for childbirth, and pregnancy complications diagnosed. The second was a seven-item section on CAM treatments and prescriptions initiated by practitioners and targeting a particular symptom or condition. The third was a 25-item section on CAM as self-care, and included; the information source (fill in response), reasons for each CAM use (fill in response), and if they consulted with their health care provider about the use of CAM. Regarding the items that we selected for both treatment and self-care, blank spaces were available for participants to list any other CAM treatments they used. In addition, we asked participants’ interest in CAM therapy or self-care during pregnancy. Because of the range and variety of CAM therapies, we chose items from the categories of natural products, mind and body practices, and other complementary health approaches that tended to have a high frequency use [4], and which had been used by pregnant women, based on the results of previous studies [14, 22, 26, 27]. A small feasibility study was conducted among eight women to ensure the questionnaire was readable, understandable, and easily to complete and we used their feedback to clarify the questionnaire. Next, we prepared a paper version and an online version of the final questionnaire so that the participants could choose which format they preferred to use.

### Data collection

Pregnant women who met the inclusion criteria were selected based on purposive sampling. The researcher or a midwife at the participating facility distributed the questionnaire to eligible women at the time of their pregnancy checkups. If they agreed to participate in this study, they were asked to complete the survey questionnaire by themselves. If women preferred the online questionnaire, they could answer it at their convenience and submit it before the end of the study. Women who chose a paper version completed it while they were waiting for the pregnancy check-up, and then placed it into the collection box, anonymously. The data were collected between September 2017 and December 2018.

### Data analysis

For data analysis, descriptive statistics such as frequencies with percentages or means with SD were calculated for all items. Bivariate analysis comparing the use and non-use of CAM by demographics was performed using chi square (χ^2^) or the Fisher’s exact test. Multivariate logistic analyses were then conducted, to clarify the factors correlated with CAM use for therapy by practitioners or self-care, using IBM SPSS Statistics 27. In the univariate logistic analysis, factors with a probability value (p) of < 0.10 in their odds ratio were entered into the multiple logistic analyses. For the other tests, the significance level was set at 5%, and a two-sided test was performed.

### Ethical considerations

This study was conducted in accordance with the Declaration of Helsinki and the Ethical Guidelines for Medical and Health Research involving Human Subjects (Ministry of Health, Labour and Welfare, 2015). St. Luke’s International University Research Ethics Review Committee provided ethical approval (17-A068).

## Results

### Participants and response rate

In this study, 580 pregnant women at five facilities were given a paper questionnaire and an explanation sheet containing the URL to respond if they chose the Internet survey. Responses were received from 394 women (response rate, 67.9%).

The demographic and obstetric characteristics of the participants are presented in Tables 1 and 2. A small majority (65.7%) of women were in their 30 s. Almost all were married, and approximately half (53.8%) were experiencing their first pregnancy. Almost all had an education beyond high school. When submitting their replies, 355 women planned to undergo vaginal deliveries and 30 women had scheduled cesarean sections. Among those scheduled for vaginal delivery, 14.0% planned to use epidural analgesia. A total of 288 (73%) pregnant women were experiencing pregnancy complications such as anemia, breach presentation, gestational diabetes mellitus or minor symptoms of threatened preterm birth.

**Table 1:**
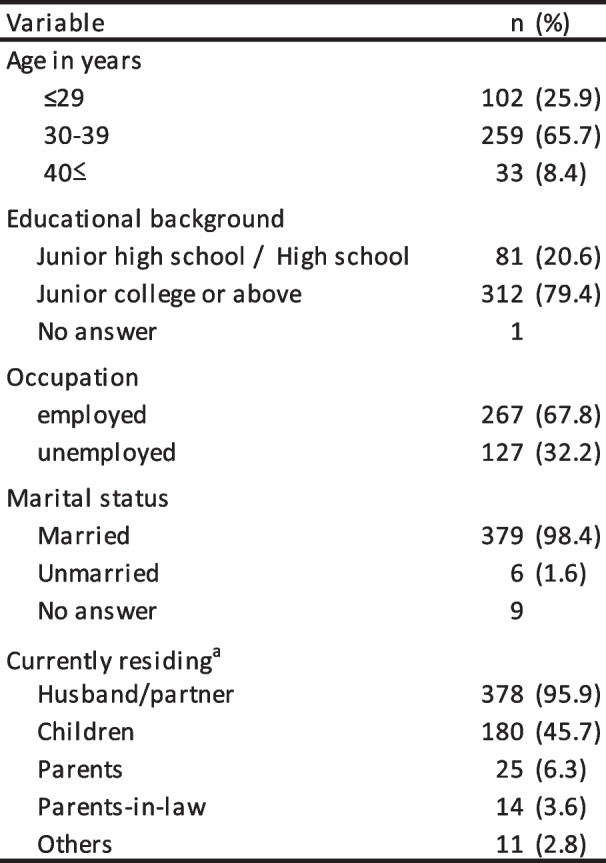
Demographic characteristics (*N* = 394)

^a^Currently residing had multiple answer options

**Table 2:**
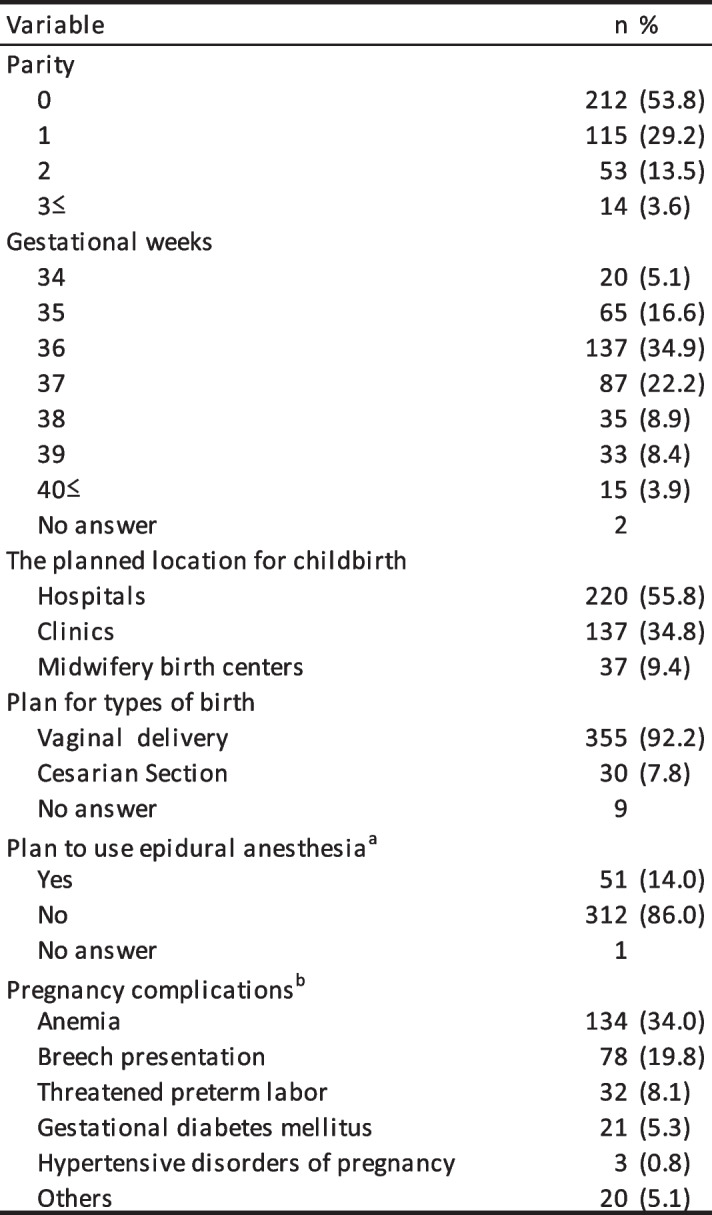
Obstetric characteristics (*N* = 394)

^a^The number of participants who planned to use epiduralanesthesia did not include those who had a plannedcaesarean section (*n* = 364)

^b^The number of participants who were experiencingpregnancy complications was 277. Participants could selectmultiple complications

### CAM therapies received by practitioner

Approximately 75 women (19.0%) answered that they received CAM therapy from their practitioner. There were multiple answers for therapies experienced (in order of most use): traditional Chinese medicine, chiropractic, moxibustion, and acupuncture.

#### Traditional Chinese medicine

Traditional Chinese Medicine was prescribed by a practitioner for 31 women (7.9%) including prescriptions for medicines such as *Rikkunshito* (“six gentlemen soup”), *Yokukansan* (“suppressing liver”), *Keishikaryukotsuboreito* (“bone oyster hot water”), and *Toki-syakuyaku-san* (Angelica and Peony powder). The purpose of the therapies was the “improvement of physical disorder or symptoms” (*n* = 16), treatment for *hiesho* (body coldness) (*n* = 7), and “reducing uterine contractions” (*n* = 2).

#### Chiropractic

Twenty-seven women (6.9%) had received chiropractic treatment as therapy at chiropractic clinics, or delivery facilities where midwives trained in maternity manipulation provided treatment. The purpose of treatment was the “improvement of physical disorder or symptoms,” including “relief of back pain” (*n* = 20), “breech presentation treatment” (*n* = 6), and “physical health management” (*n* = 1).

#### Moxibustion

Moxibustion was received as a treatment by 25 women (6.4%), either by an acupuncturist at a moxibustion and acupuncture clinic, or at a delivery facility which had an acupuncturist. The purpose of the treatment was “breech presentation treatment” (*n* = 13), “improvement of physical disorder or symptoms” (*n* = 6), and “warming body” (treating *“hiesho”*) (*n* = 4).

#### Acupuncture

Twenty-one women (5.3%) who received acupuncture were treated mainly at an acupuncture or moxibustion clinic or by an acupuncturist at the delivery facility. The purpose of the treatment included, “improvement of physical disorders or symptoms” including lumbago (low back pain) (*n* = 10), and “breech presentation treatment” (*n* = 9).

### CAM use as self-care

**Fig. 1:**
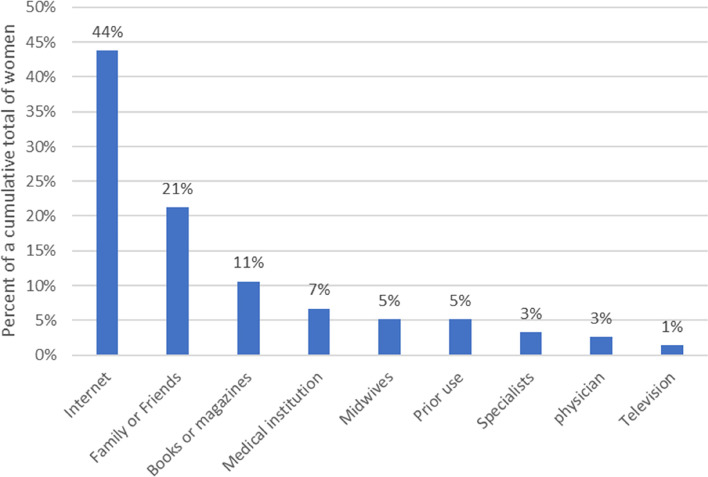
Information sources for complementary and alternative medical as self-care. Note. The number is a cumulative total of women who used CAM as self-care with multiple answer (*n* = 944)

There were 348 women (88.3%) who reported using at least one form of CAM as self-care during pregnancy. Table 3 shows the frequencies for each type of CAM used by 30 women or more. The resources used for obtaining information regarding CAMs are displayed in Fig. 1. The “Internet” was the most common, followed by “family or friends”, “books or magazines”and “medical institution (antenatal class, material, etc.)”. Very few health care providers were a resource for CAM information.

**Table 3:**
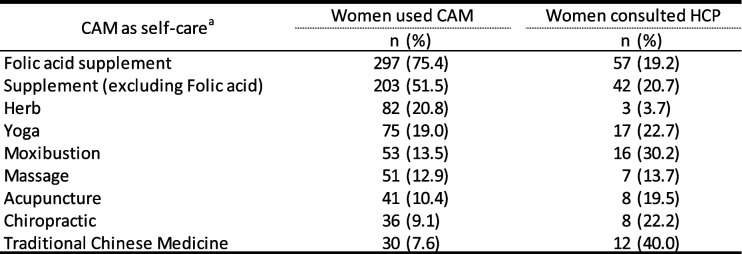
Complementary and alternative medical use as self-care by type and use ofconsultation (*N* = 394)

*CAM* complementary and alternative medicine, *HCP* health care provider

^a^Particpants could select multiple CAMs

#### Natural products

The most commonly used CAM among pregnant women was folic acid supplementation, used by 75.4% of women. Supplements (excluding folic acid supplements) were next, used by 203 women (51.5%); among these, 20.7% of women consulted the health care provider about their use. The main reasons for use were “preventing anemia” (*n* = 29), “fetal health” (*n* = 21), and “as nutritional supplementation” (*n* = 21). Various specific supplements were used: “iron” (*n* = 41), “vitamins” (*n* = 13), “calcium” (*n* = 11), and “DHA” (*n* = 6). Herbs (in the form of herbal tea) were used by 20.8% of women during pregnancy, and only three (3.7%) consulted the health care provider about their use. The main reason was for a “safe birth” (*n* = 30), “alternative to caffeine-containing beverage” (*n* = 17), and “improvement of ‘*hiesho*’” (*n* = 16). Various types of herbs, such as “raspberry leaf” (*n* = 28) and “rooibos” (*n* = 19), were used.

#### Mind and body practices

Concerning mind and body practice, Yoga was the most popular, and it used by 19.0% of women. The main reasons stated were “fitness training” (*n* = 33), “relaxation” (*n* = 11), and “safe birth” (*n* = 7). Moxibustion was used by 13.5% of women, 30.2% of whom consulted with health care provider. The specific application was soft moxibustion in the lower calf area. The main reasons were: “improvement of physical disorder,” including “*hiesho*” and back pain (*n* = 24); “breech presentation” (*n* = 10); “to keep pregnancy healthy” (*n* = 5); “for safe childbirth” (*n* = 2); and “others” (*n* = 6).

Massage was used by 12.9% of women for: “improvement of back pain” (*n* = 19); “improvement of edema of the legs” (*n* = 8); “improvement of stiff shoulder” (*n* = 4); and “relaxation” (*n* = 4). Acupuncture was used by 41 women (10.4%), and eight (19.5%) had consulted health care provider. Specific applications included self-applied patch acupuncture. The main reasons were: “improvement of physical disorders,” including body stiffness and back pain (*n* = 20); “to keep pregnancy healthy” (*n* = 6); “breech presentation” (*n* = 4), “safe birth” (*n* = 1), and “others” (*n* = 3). Chiropractic was used by 9.1% of women. The reasons were as follows: “improvement of physical disorder,” including back pain (*n* = 24); “to cure distortion of the body and pelvis” (*n* = 4); “for physical condition management” (*n* = 2); and “other reasons” (*n* = 6).

### Other complementary health approaches

Traditional Chinese medicine was used by 30 women (7.6%), 40.0% of whom consulted a health care provider regarding its use. Specific uses included the popular *Kampo* (Chinese) medicine “*Toki-shakuyaku-san* (TJ-23).” The reasons were: “aid for pregnancy/ infertility treatment” (*n* = 11); “physical disorder,” including “*hiesho*,” (*n* = 8); and “others” (*n* = 2).

### Characteristics of participants and CAM use

Tables 4 and 5 show demographic characteristics of women and the association with CAM. The utilization rate of CAM therapies received via a practitioner differed depending on planned location for childbirth (hospital, 16.8%; clinic, 16.9%; midwifery birth center, 40.5%) and whether or not there were pregnancy complications (complication, 24.2%; no complication, 12.0%). On the other hand, the utilization rate of CAM as self-care differed depending on age (≤ 29 years, 73.5%; 30–39 years, 90.4%; > 40 years, 87.9%), educational background, 70.4% for junior high or high school graduates and 89.7% for junior college or above graduates, birth history (parity, 91.0% of primiparas and 79.7% of multiparas). The utilization rate of CAM for self-care also varied depending on whether the mother planned to use epidural analgesia (85.3%) or not (96.1%).

**Table 4:**
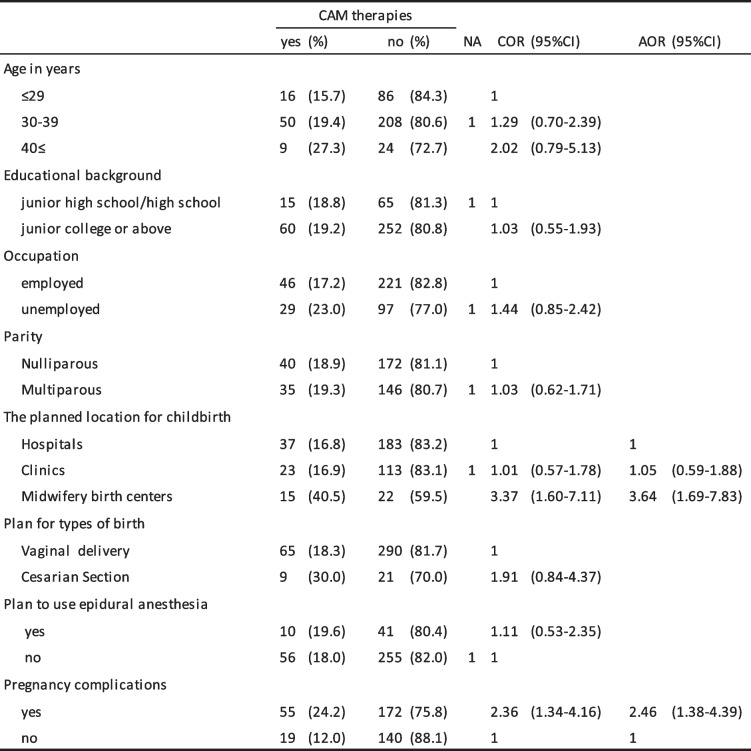
Demographic characteristics of pregnant women and the association with CAM received as therapies

*NA* no answer, *COR* crude odds ratio, *AOR* adjusted odds ratio

**Table 5:**
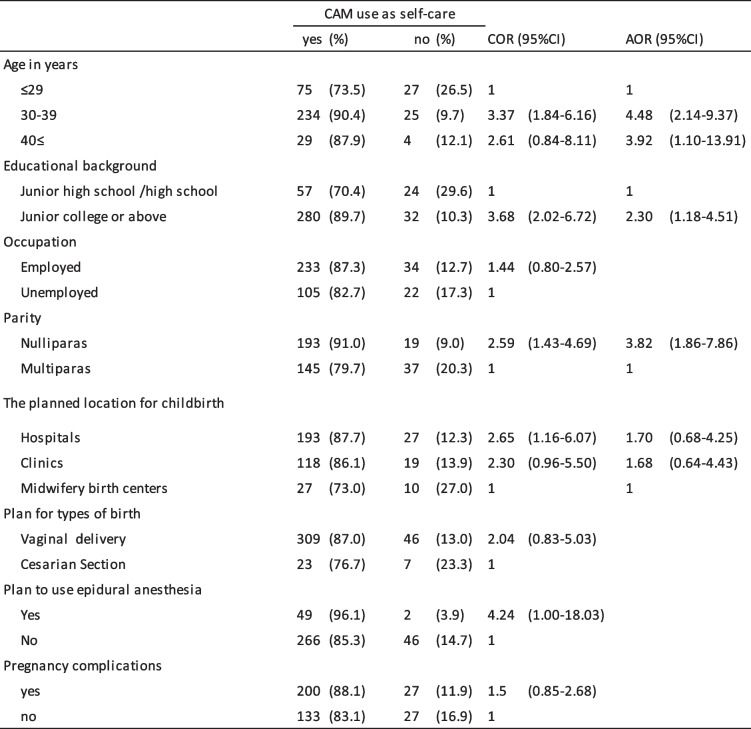
Demographic characteristics of pregnant women and the association with CAM use as self-care

*COR* crude odds ratio, *AOR* adjusted odds ratio

Multiple logistic regression analyses revealed that the factors associated with CAM therapy by practitioners were midwifery birth centers in planned childbirth settings (adjusted odds ratio [AOR] 3.64, 95%CI [1.69–7.83]; reference hospital), and pregnancy complications (AOR 2.46, 95%CI [1.38–4.39]). The factors related to use of CAM as self-care were age 30–39 years (AOR 4.48, 95%CI [2.14–9.37]; reference < 30), junior college or above (AOR 2.30, 95%CI [1.18–4.51]), and primiparas (AOR 3.82, 95%CI [1.86–7.86]).

### Interest in therapy offered by CAM

When asked about their interest in CAM therapies during pregnancy, the most common responses were: massage 212 (53.8%), yoga 209 (53.1%), folic acid supplement 186 (47.2%), aromatherapy 181 (45.9%), and relaxation 162 (41.1%) which could be incorporated into daily life as a self-care. Moreover, 147 women (37.3%) were interested in chiropractic, 108 (27.4%) in moxibustion, 100 (25.4%) in acupuncture, and 84 (21.3%) in traditional Chinese medicine which would need a practitioner consultation.

## Discussion

The present study constitutes a pilot survey of CAM use by pregnant women. Since this is the first study to clarify the utilization of CAM in Japan, the questionnaire was original, developed by the researchers. Importantly, we identified utilization of CAM treatment by practitioners and CAM as self-care including information source and reasons.

### Utilization of CAM during pregnancy

In this study, whereas about 20% of pregnant women were prescribed CAM use, almost 90% of those responding used some type of CAM, independently, as self-care, which is higher than found in other studies. Previous reviews reveal, a wide variation in the use of CAM. In literature reviews, the prevalence rates of CAM for self-care ranged from 1 to 87% [5, 21] and from 6% to74% [22]. Although the definitions of CAM, research designs and time frames of pregnancy differed among studies, it is clear that in Japan CAM use is common.

There are several reasons why the prevalence of use of CAM in this study in Japan is so high. One that seems obvious is that CAM use has become more widespread with the advent of the Internet. In addition, the most frequent CAM use was intake of folic acid as a dietary supplement and taking folic acid supplement to prevent neural tube defects is recommended based on the widespread use of the 2014 [28] and 2020 Japan Society of Obstetrics and Gynecology (JSOG) and Japan Association of Obstetricians and Gynecologists (JAOG) [29] guidelines. In addition, women reported various pregnancy-related symptoms which may have resulted in seeking the use of CAM for the treatment for each symptom. One study found that pregnant women believed that CAM was not harmful to them or their babies during pregnancy and being able to select a CAM treatment without the health care provider’s permission gave them more control over their health/body [15]. In fact, in many cases, research indicates that these symptoms could be resolved with treatments other than conventional medicine [30], therefore, pregnant women may assume that CAM options are effective and safe choices to alleviate discomfort. Furthermore, prior studies found that midwives thought CAM was a useful and important aspect of healthcare during pregnancy [31, 32]. There is a 2020 Japan Academy of Midwifery evidence-based clinical guideline that provides recommendations about the use of some CAM options during pregnancy [33]. There is a strong possibility that midwives recommend various CAM options for pregnant women. However, it is not known the extent to which Japanese midwives are using the clinical guidelines and would benefit from further research. In the meantime, it remains a necessity to educate midwives and healthcare providers about safe and evidence-based use of CAM.

### Relationship between characteristics and utilization

In this study, three characteristics were associated with CAM utilization as self-care: age, academic background and parity and is similar with previous surveys [5, 21, 22]. With regard to educational background, it is unlikely that CAM is learned or experienced at high school or university levels. However, as the academic level rises, the ability and capacity to gather information most likely increase, along with the ability to critically appraise the information. In our survey, nulliparous women used CAMs more compared to multiparas. It is reported that nulliparous women tended to engage favorable health practice during pregnancy [34, 35]. Using CAM as self-care might be one of the good health practices. Also, nulliparas are more likely to have concerns and worries for pregnancy and childbirth [36–38]. In addition, a limiting factor for multiparous women is the cost and time required for their implementation. Money and time are needed to take care of older children; therefore, another factor is that their time spent alone is limited.

In terms of CAM therapies provided by practitioners, planned location for childbirth was related factor. The utilization rate of CAM therapies was higher among women who planned to deliver at midwifery birth centers than was that in those who planned to deliver at other institutions. Midwives who work at midwifery birth center prioritize prevention [39], as their scope of practice does not include medical procedures or prescribing medications. In addition, midwives have a positive view on the usefulness of CAMs in general [31, 32]. Women who have complications such as breech presentation may seek a CAM by a practitioner as there is evidence that supports the effectiveness of moxibustion and acupuncture for breech presentation [40]. Thus, as an alternative, midwives tended to recommend CAM use as a treatment to prevent and alleviate physical and mental disorders associated with pregnancy while remaining within their scope of practice. Women who choose midwifery birth centers value choice and control [41] or avoidance of unnecessary interventions [42, 43].

### Information sources for CAM utilization

Many pregnant women used the “Internet” as an information source in this survey, and this result was similar to that of previous studies [44–46]. In a United States study, women mainly used the Internet to search “sites run by health professionals” and “government sites” [46]. To date, few reliable sites in Japan have written about CAM use and its evidence for pregnant women [47]. Therefore, we must create Internet sites so that pregnant women can obtain information and choose safe and suitable CAM. At its most basic, women's health literacy skills need to be improved. Even those women with high health literacy skills, which led them to engage in an information-seeking and analysis process before making the decision to use CAM or not still need accurate information [48]. Therefore, providing practical and safe information about CAM through the Internet and providing women with high health literacy skills are both essential for health of pregnant women and their baby.

In addition, positive therapeutic relationships with health care practitioners are necessary for decision making to use CAM [49]. Health care practitioners can enhance their interactions with pregnant women who use CAM by respectfully discussing use within the context of these women's values and health goals [50]. However, healthcare providers as information sources were limited in Japan. In fact, in this study only about 19% of women consulted health care providers about use of CAM. A United Kingdom study found that disclosure to medical personnel was approximately 70% [14], and nearly 90% in Australia and New Zealand [51], which is certainly higher than the results of this study. Health care providers in Japan should consider collecting daily communications from pregnant women who are using or considering using CAM therapies, and then verify whether the information and knowledge necessary for determining its use are insufficient or incorrect in order to grasp and provide information. Additionally, health care providers need to be more proactive in providing information using opportunities of pregnancy check-up and consultation particularly with primiparas.

It is necessary for health care providers to deepen their interest in and knowledge of CAM in order to use it effectively. However, currently, there are few opportunities for CAM education and practice for medical staff in Japan. Moreover, midwives believed that they should have more knowledge about CAM [31]. Thus, we surmise that a future goal for health care providers is to build a system that provides comprehensive education, evidenced-based clinical guidelines and a practical system that is not limited to researches.

#### Interest in pregnant women’s CAM care

To date, almost no studies in Japan have assessed pregnant women’s interest in CAM. To promote education and research in the future, we hope this survey can be a source of information for determining the priorities and needs for selecting therapies. Further, it is also important to adopt a more detailed perspective from a qualitative approach, as used in previous research [52, 53], on participants’ awareness of CAM.

### Limitations of this study

This study has several limitations. The questionnaire was developed by researchers and would benefit from psychometric testing to improve validity and reliability. The survey achieved a 68% response rate. Currently, typical Japanese surveys have a response rate between 50–60%, and the result of an analysis of bias and responses rates indicates that the potential for bias is relatively low [25]. However, participants of this study were purposively selected, which is prone to bias. A random sampling technique would reduce that bias. Another limitation of concern was that six out of seven facilities cooperating with this study were in the Kanto district of Japan. This district contains several densely populated, highly urbanized cities (i.e., Tokyo and Yokohama); therefore, the survey results may contain a regional bias. Therefore, future studies should focus on wider geographic areas. Findings should be interpreted cautiously while the limitations provide ample direction for future research.

## Conclusions

Approximately 20% of Japanese pregnant women received CAM as therapy by practitioners, and the related factors were: tended to have baby at midwifery birth center and pregnancy complications. Almost 90% of respondents used CAM as self-care and the related factors were: older, had a higher educational level and tended to be primiparas. The Internet is the more common source of information regarding CAM rather than health care providers. Health care providers need to provide evidenced-based information on CAM and to help decision making to ensure safe and effective CAM utilization by pregnant women. While the self-report questionnaire shows great promise in providing useful data, it needs validity and reliability testing with a range of pregnant women from various regions in Japan. After which, full-scale studies should be conducted.

## Data Availability

The datasets used and/or analyzed during the current study are available from the corresponding author on reasonable request.

## Abbreviations

AOR: Adjusted odds ratio
CAM: Complementary and alternative medicine
CI: Confidence interval
COR: Crude odds ratio
NCCIH: National center for complementary and integrative health
QOL: Quality of life
